# Hox gene cluster of the ascidian, *Halocynthia roretzi*, reveals multiple ancient steps of cluster disintegration during ascidian evolution

**DOI:** 10.1186/s40851-017-0078-3

**Published:** 2017-09-15

**Authors:** Yuka Sekigami, Takuya Kobayashi, Ai Omi, Koki Nishitsuji, Tetsuro Ikuta, Asao Fujiyama, Noriyuki Satoh, Hidetoshi Saiga

**Affiliations:** 1 Department of Biological Sciences and Technology, Tokyo Metropolitan University, 1-1 Minamiohsawa, Hachiohji, Tokyo, 192-0397 Japan; 2 Marine Genomics Unit, Okinawa Institute of Science and Technology Graduate University, Onna, Okinawa, 904-0495 Japan; 3 National Institute of Genetics, 1111 Yata, Mishima, Shizuoka, 411-8540 Japan

**Keywords:** Hox gene cluster, Ascidian, Tunicate (urochordate) evolution, *Halocynthia roretzi*

## Abstract

**Background:**

Hox gene clusters with at least 13 paralog group (PG) members are common in vertebrate genomes and in that of amphioxus. Ascidians, which belong to the subphylum Tunicata (Urochordata), are phylogenetically positioned between vertebrates and amphioxus, and traditionally divided into two groups: the Pleurogona and the Enterogona. An enterogonan ascidian, *Ciona intestinalis* (*Ci*), possesses nine Hox genes localized on two chromosomes; thus, the Hox gene cluster is disintegrated. We investigated the Hox gene cluster of a pleurogonan ascidian, *Halocynthia roretzi* (*Hr*) to investigate whether Hox gene cluster disintegration is common among ascidians, and if so, how such disintegration occurred during ascidian or tunicate evolution.

**Results:**

Our phylogenetic analysis reveals that the *Hr* Hox gene complement comprises nine members, including one with a relatively divergent Hox homeodomain sequence. Eight of nine *Hr* Hox genes were orthologous to *Ci-Hox1*, *2, 3, 4, 5, 10, 12* and *13.* Following the phylogenetic classification into 13 PGs, we designated *Hr* Hox genes as *Hox1, 2, 3, 4, 5, 10, 11/12/13.a*, *11/12/13.b* and *HoxX*. To address the chromosomal arrangement of the nine Hox genes, we performed two-color chromosomal fluorescent in situ hybridization, which revealed that the nine Hox genes are localized on a single chromosome in *Hr*, distinct from their arrangement in *Ci*. We further examined the order of the nine Hox genes on the chromosome by chromosome/scaffold walking. This analysis suggested a gene order of *Hox1*, *11/12/13.b, 11/12/13.a, 10, 5, X,* followed by either *Hox4, 3, 2* or *Hox2, 3, 4* on the chromosome. Based on the present results and those previously reported in *Ci*, we discuss the establishment of the Hox gene complement and disintegration of Hox gene clusters during the course of ascidian or tunicate evolution.

**Conclusions:**

The Hox gene cluster and the genome must have experienced extensive reorganization during the course of evolution from the ancestral tunicate to *Hr* and *Ci*. Nevertheless, some features are shared in Hox gene components and gene arrangement on the chromosomes, suggesting that Hox gene cluster disintegration in ascidians involved early events common to tunicates as well as later ascidian lineage-specific events.

**Electronic supplementary material:**

The online version of this article (10.1186/s40851-017-0078-3) contains supplementary material, which is available to authorized users.

## Background

Hox genes comprise a subset of Antp class homeobox genes [1] conserved throughout animal phylogeny, and are closely involved in morphogenetic patterning along the anterior-posterior axis. In chordates, Hox genes are classified into 13 paralog groups (PGs) according to homeodomain similarity [2]. Hox genes are often found in a relatively narrow region on one chromosome, forming a Hox gene cluster. It is generally accepted that the Hox gene cluster consists of a subset of the 13 PG Hox genes, which are aligned according to PG number, and that have the same transcription direction, spanning about 100–120 kb on a chromosome. These characteristics of the Hox gene cluster are almost exclusively observed in vertebrate genomes [3]. In contrast, invertebrate Hox gene clusters span the chromosome much more broadly than do their vertebrate counterparts [3], though the number of Hox genes that constitute a single cluster in invertebrates is at most 13, with the exception of amphioxus and lepidopteran insects, which possess 15 [4, 5] and 14 or more Hox genes [6], respectively. It is now accepted that Hox gene clusters in vertebrates are exceptionally tightly organized and that the structure of the Hox gene cluster, or the placement of Hox genes on chromosomes, is more variable among invertebrates [3]. The biological significance of such Hox gene clustering in a relatively small portion of the genome has only been explained to a certain extent in vertebrates, while in other taxa it remains poorly understood [3].

Ascidians belong to the class Ascidiacea, subphylum Tunicata (Urochordata), and phylum Chordata [7]. Ascidians occupy a phylogenetic position between vertebrates and amphioxus [8, 9]. Amphioxus, a basal chordate, has a single Hox gene cluster, consisting of 15 Hox genes with the same transcription direction, spanning about 470 kb [4]. In the ascidian, *Ciona intestinalis* (*Ci*), nine Hox genes were identified during draft genome analysis [10]. It was subsequently revealed that the nine *Ci* Hox genes are on two chromosomes, seven on one and two on the other, exhibiting an unusual gene order as shown using chromosomal FISH analysis by our group [11]. Based on these observations, it was suggested that the Hox cluster has disintegrated in *Ci*, with the loss of some genes and changes in gene placement on the chromosome [12]. It is thus anticipated that the Hox gene cluster may have also disintegrated in other ascidians. However, substantial evidence to support this speculation has yet to be reported. If such disintegration did in fact occur, the process responsible for its occurrence in ascidian evolution remains enigmatic. In the present study, we address these points.

Ascidians are traditionally divided into two groups (subclasses) [7], Enterogona (Aplousobranchia and Phlebobranchia) and Pleurogona (Stolidobranchia). *Ci* is a member of the Phlebobranchia, and *Hr* belongs to the Stolidobranchia. Both species are widely used in scientific research, especially in developmental studies, and their embryos exhibit very similar development, including most of the same cell lineages [7]. Nevertheless, the non-protein coding regions of their genomes are difficult to align [13], reflecting a remote phylogenetic relationship between these two ascidians.

In the present study, we analyzed the Hox gene complement and its organization in the *Hr* genome by chromosomal FISH and chromosome walking to clarify the Hox gene cluster structure. We show that the Hox gene complement consists of nine genes in *Hr,* as in *Ci*, but unexpectedly, the nine Hox genes of *Hr* reside on a single chromosome. We further inferred the Hox gene order on the chromosome. By comparing the information of *Hr* with that of *Ci* as well as of other lower chordates, we propose a scenario to explain how the disintegration occurred during ascidian or tunicate evolution.

## Methods

### Isolation of *Halocynthia roretzi* genomic DNA and Hox gene candidates

*Halocynthia roretzi* genomic DNAs were prepared individually from single adult animals. Gonads were excised and frozen in liquid nitrogen. Genomic DNA was prepared according to the protocol of Blin and Stafford [14] and used for PCR as templates. We found that genomic PCR was occasionally not successful with some DNA preparation, probably due to genomic sequence heterogeneity among individuals.

Genomic PCR for isolation of Hox gene candidates was performed as described previously [15]. Additionally, the following degenerate primer sets were used: 5′GARYTNGARAARGARTTY3′ (corresponding to ELEKEF), 5′AARAARMGNCARCCNTAY3′ (KKRQPY) and 5′NCKNCKRTTYTGRAACCA3′ (WFQNRR).

### Phylogenetic analysis of *Halocynthia roretzi* Hox gene candidates

Phylogenetic analysis of Hox gene candidates was done using CLUSTAL W for alignment and the MEGA5 software package [16] to construct ML trees with 1000 trials. For the reference data set, homeodomains with the flanking 20 N-terminal and seven C-terminal amino acid residues (87 amino acid residues) of 39 mouse (*Mus musculus*), 35 coelacanth (*Latimeria menadoensis*), 21 horn shark (*Heterodontus francisci*) and nine ascidian (*Ciona intestinalis; Ci*) Hox proteins were used (see Additional file 1: Figure S1).

### Construction of a BAC library of *Halocynthia roretzi*

A *Halocynthia roretzi* BAC library was constructed from sperm DNA of a single adult *H. roretzi*, which was obtained at Otsuchi Marine Research Center of the University Tokyo, in Iwate, Japan.

A BAC library was constructed essentially as described previously [17]. The sperm was washed twice with phosphate-buffered saline and then with lysis buffer (10 mM Tris-HCl pH 8.0, 50 mM NaCl, 1% lithium dodecyl sulfate, 100 mM EDTA) for 2 h at 37 °C in a 1.0% agarose gel plug. The plug was stored in 20% NDS solution (0.2% N-lauryl sarcosine, 2 mM Tris-HCl pH 9.0, 0.14 M EDTA). After exchanging the NDS buffer with TE, genomic DNA was digested partially with *Bam*H1, and 150–250 kb DNA fragments were isolated using pulsed field gel electrophoresis. DNA fragments eluted from the gel were ligated into pKS145 and an aliquot of the ligation reaction mixture was used to transform *E. coli* DH10B. Using a Flexys robot (Genomic Solutions, USA) and a 3D:Biomek FX robot (Beckman Coulter, USA), a total of 20,736 BAC clones were picked up and arrayed in 54 × 384-well microtiter plates in LB medium containing 10% glycerol and 25 μg/mL ampicillin. Plates were incubated overnight at 37 °C and then stored at –80 °C. The BAC library was constructed so as to be amenable to the dimension pooling system for PCR screening.

To estimate the size of the average genomic DNA insert in the BAC library, 20 randomly selected clones were digested with *Not*I and analyzed by pulsed field gel electrophoresis. This analysis revealed that the average insert size was 110 kbp, and the coverage was estimated as 14.2 ×, assuming that the genome size of *H. roretzi* is 160 Mbp [13].

### Embryos for chromosomal FISH (fluorescence in situ hybridization)

Fertilized eggs were raised in FSW until the 2-cell stage. When embryos began to divide to the 4-cell stage, they were transferred into Ca^++^ free seawater. When they reached the 64-cell stage, colchicine (Sigma) was added at a final concentration of 0.025% (*w*/*v*). Embryos were cultured for 30 min and fixed with acetic acid:methanol (3:1) overnight, and then transferred to 70% ethanol and kept at –20 °C until use.

### Fluorescence in situ hybridization (FISH)

Two-color chromosomal FISH was performed according to a procedure previously described for *Ci* with some modifications [11]. For preparation of metaphase spreads, 15–30 fixed embryos were de-chorionated manually under a stereomicroscope and embryonic cells were transferred to a microtube. After removal of excess liquid, 100 μL of 60% acetic acid was added to the tube, and the cell suspension was mixed by gentle rolling for 90 s. Then, the mixture was agitated for 30 s by gentle pipetting about 20 times. Immediately after agitation, the mixture was spread gently on a warmed (48 °C) clean slide glass using a pipet. The glass slide was allowed to stand at 48 °C for 2.5 h before being subjected to FISH. Probes for chromosomal FISH were prepared using BAC clones labeled with biotin or digoxigenin using a nick translation kit (Roche).

### Chromosome/scaffold walking with BAC library and ANISEED database

Chromosome walking using PCR and BAC library screening was performed using standard methods [18, 19]. Scaffold walking is here referred to as a modification of chromosome walking, using nucleotide sequence information of scaffolds out of database open to public for designing PCR for BAC library screening to identify adjacent scaffolds.

A pair of primers was designed around the starting BAC clone end region. The primers were used for PCR screening of the BAC library. When positive clones were found, their DNAs were prepared and sequenced from both ends of the insert. The resulting nucleotide sequences were used for designing PCR primers. By using the primers and DNAs of isolated clones and of the starting BAC clone, reciprocal PCR was performed to determine the relative positional relationship between isolated clones and the end region of the starting BAC clone, and a desired clone was identified. By using the identified clone, another screening cycle was performed to walk further along the chromosome.

In the case of scaffold walking, the resulting nucleotide sequences were used for BLAST surveys of the *Halocynthia roretzi* genomic sequences, Halocynthia roretzi MTP2014, of the ANISEED genomic database [20] (https://www.aniseed.cnrs.fr/aniseed/default/blast_search) to locate the ends of the BAC clone on scaffolds. A pair of primers was designed in the end region of the scaffold, tested for compatibility with PCR using several genomic DNA preparations, and used for the screening of the BAC library. When the positive clones were isolated, the nucleotide sequences were determined for both end regions, and the resulting nucleotide sequence information was used for the BLAST surveys to identify an adjacent scaffold so as to walk further along the chromosome.

### Nucleotide sequence determination of the BAC clone end regions

BAC clone DNA was prepared from 5 mL overnight culture using a QIAprep Spin Miniprep kit (QIAGEN). The BAC end-region sequence was determined by a standard method using BigDye ver. 3, upper and lower primers for pKS145 vectors and ABI 3000 sequencers.

### Nucleotide sequence determination of a BAC clone insert

Circular BAC clone DNA was prepared using a QIAGEN Large-Construct kit according to the supplier’s procedure, in which an exonuclease digestion procedure is included (QIAGEN). For nucleotide sequence determination of BAC clone DNAs, an Illumina Miseq was used. Libraries were prepared according to a protocol provided by the manufacturer, with slight modifications. Fragmented BAC clone DNA was further purified using Blue Pippin (Sage Science). A paired-end library consisting of clones containing ∼720 bp insert DNA fragment was prepared for the Miseq using a TruSeq DNA PCR-Free LT Sample Prep Kit (Illumina). Adapter sequences were removed from all sequence reads using Trimmomatic-0.30 [21]. Paired-end reads of high quality (quality-value ≥20) were assembled de novo using Newbler 2.9 (GS Assembler) to create a scaffold. From the scaffold, vector sequence was removed, and genomic sequence was extracted.

## Results

### Hox gene complement in *Hr* genome

In a previous study, we reported the isolation of Hox1, as well as Hox gene fragments from the *Hr* genome. Hox1 was identified by alignment to other homeodomain sequences available at that time, and its structure and developmental expression were reported [15]. Since then, we have repeated genomic PCR with various sets of degenerate primers and RT-PCR, using RNA from embryos at various stages, and eventually isolated nine Hox gene candidate sequences, including the previously reported Hox1 from the *Hr* genome. These candidates were subjected to phylogenetic analysis in the present study.

**Fig. 1:**
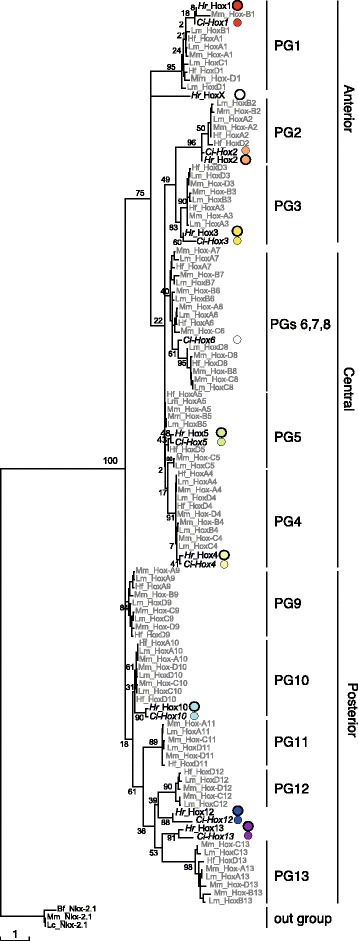
Phylogenetic analysis of Hox gene candidates of *Halocynthia roretzi*. The ML tree was constructed using homeodomain sequences and the adjacent 20 N-terminal and seven C-terminal amino acid residues (Additional file 1: Figure S1) and MEGA5 software package. The percentage of 1000 replicated trees in which clustering of genes was supported is indicated at nodes. Within a clade consisting only of vertebrate Hox genes, the percentage is not indicated. *Hr* Hox gene candidates and Hox genes of *Ciona intestinalis (Ci-Hox)* are indicated by larger and smaller colored circles, respectively. Here, *Hr* Hox gene candidates are tentatively designated according to their *Ci* counterparts, except for HoxX, which did not show apparent orthology to *Ci-Hox* genes. The color-code indicates distinct paralog groups (PGs). Taxonomic abbreviations are Mm for *Mus musculus*, Lm for *Latimeria menadoensis*, Hf for *Heterodontus francisci*, Ci for *Ciona intestinalis* and Hr for *Halocynthia roretzi*. The bar at the bottom indicates one amino acid substitution per position in the sequence

A phylogenetic tree (Fig. 1) identified nine *Hr* Hox gene candidates (see also Additional file 1: Figure S1). Eight of the nine *Hr* Hox genes always clustered with *Ci* Hox genes, *Ci-Hox1, 2, 3, 4, 5, 10, 12* and *13*. This suggests that eight *Hr* Hox genes and their respective counterparts in *Ci* are orthologous. The remaining *Hr* Hox gene candidate did not show significant similarity to *Ci-Hox6* or any other *Ci* Hox gene. Accordingly, we tentatively designated the nine *Hr* Hox genes as *Hr* Hox1, 2, 3, 4, 5, 10, 12, 13 and X.

**Fig. 2:**
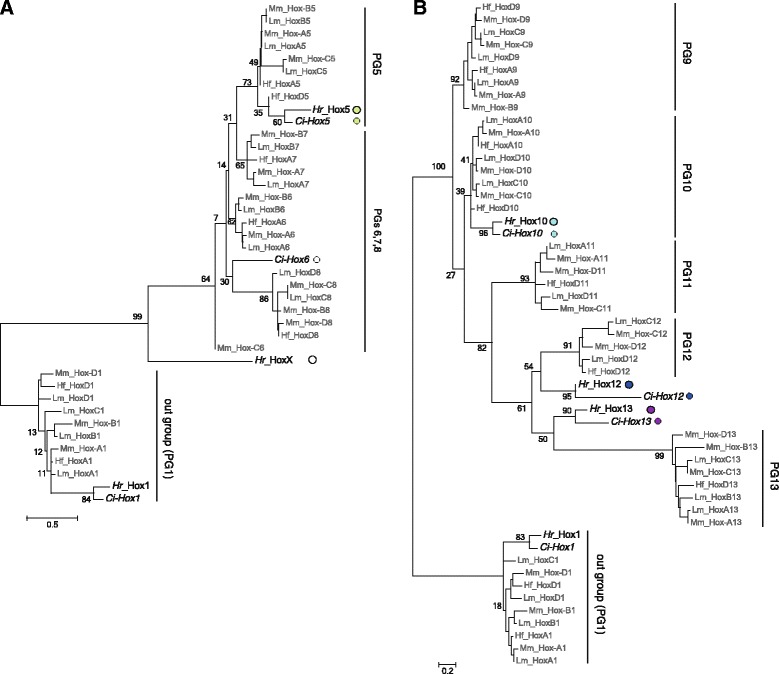
Phylogenetic analysis of Hox gene candidates of *Halocynthia roretzi*, using PGs 5–8 **a** and PGs 9–13 **b** genes, and PG1 genes were used as an out group. The ML tree was constructed using homeodomain sequences and the adjacent 20 N-terminal side and seven C-terminal side amino acid residues (see Additional file 1: Figure S1) with MEGA5 software and 1000 replicates. The percentage of replicated trees in which the clustering of genes was supported is indicated at the nodes. Within a clade consisting of only vertebrate Hox genes, the percentage was not indicated at the node. Colored circles to indicate ascidian genes and taxonomic abbreviations are the same as in Fig. 1. The bars at the bottom indicate amino acid substitutions per position in the sequence

Next, we asked to which of the 13 paralog groups (PGs) the nine *Hr* Hox genes belong. In the phylogenetic tree, *Hr* candidates for Hox1, Hox2, Hox3, and Hox4 were clearly classified into PGs 1, 2, 3 and 4 with bootstrap values of 95%, 96%, 83% and 91%, respectively (Fig. 1). Although *Hr* Hox5, 10, 12, 13 and X could not be classified into single PGs in this tree, a clade consisting of Hox genes of PGs 1–8 was supported by bootstrap values of 75% (Fig. 1); both Hox5 and X genes may thus be classified into PGs 1–8. Since Hox genes of PGs 1–4 were clearly identified, another tree was constructed to determine to which PGs the remaining two Hox genes could be classified. In a tree using Hox genes of PGs 5–8, *Hr* Hox5 and *Ci-Hox5* were likely grouped into PG5 with a bootstrap value of 73% (Fig. 2a). By contrast, HoxX was hardly classified into any PG (Fig. 2a). It is also noted here that *Ci-Hox6* could hardly be classified into PG6 (see Discussion).

Regarding the three posterior genes, Hox10 was classified into PG10, and Hox12 and 13 into PGs 11–13, albeit with poor bootstrap support (Fig. 1). Assignment of Hox10 as PG10 gene was supported by the conservation of four diagnostic residues (Gly 1, Glu 29, Leu 32, and Asp 42) in the homeodomain and three Lys residues in the flanking C-terminal side region (Additional file 1: Figure S1). These criteria were previously used to assign *Ci-Hox10* as such [22]. The remaining two posterior genes were in a clade consisting of Hox genes of PGs11–13 with relatively low bootstrap values (61%, Fig. 1). In another tree using Hox genes of PGs 9–13, the clustering of the two genes in the clade consisting of PGs 11, 12, and 13 was supported by a bootstrap value of 82% (Fig. 2b). Therefore, we propose *Hr* Hox11/12/13.a and Hox11/12/13.b as counterparts of *Ci-Hox12* and *Ci-Hox13*, respectively.

Thus, the *Hr* Hox gene complement consists of nine members. These Hox genes are designated *Harore Hox1*, *Hox2*, *Hox3*, *Hox4*, *Hox5*, *Hox10*, *Hox11/12/13.a* and *Hox11/12/13.b* according to the newly proposed nomenclature for ascidian genes [23], and the remaining one gene is tentatively designated *Harore HoxX*.

### Hox gene cluster structural analysis using chromosomal FISH

In order to address the genomic organization of the nine Hox genes using chromosomal FISH, we screened the BAC genomic library developed from sperm of a single individual and obtained clones for all nine *Hr* Hox genes. Among the clones isolated, clones containing as many as three Hox genes, except for Hox1, were found (data not shown, see next section). Using the isolated BAC clones for probes, we carried out FISH on chromosome spreads prepared from cleavage stage *Hr* embryos.

**Fig. 3:**
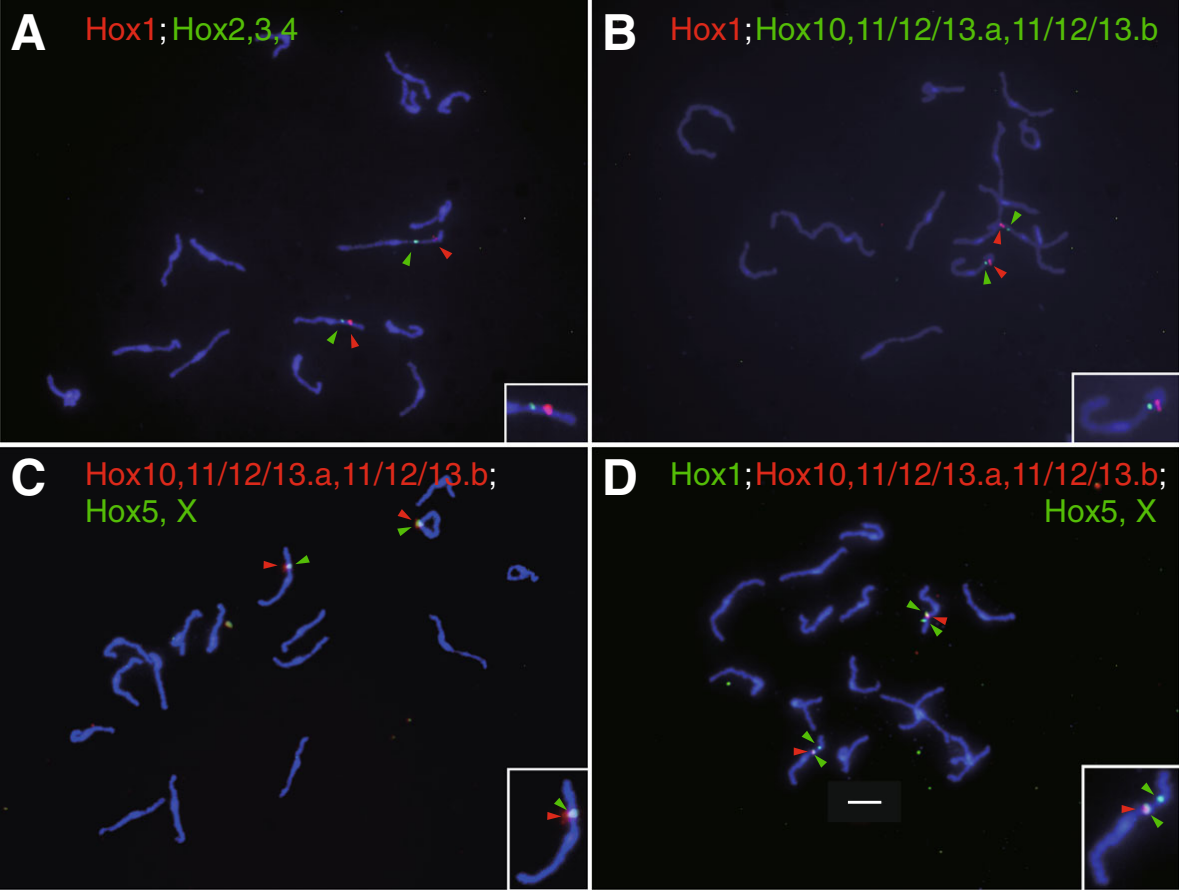
Mapping of *Halocynthia roretzi* Hox genes onto metaphase chromosomes (**a**-**d**). Metaphase chromosome spreads were prepared from cleavage stage *Hr* embryos and hybridized with two or three probes labeled with digoxigenin (red) or biotin (green) for genes indicated at the top of each panel. Chromosomes were stained with DAPI. Red and green arrowheads indicate signals for the gene of the same color code. In the right bottom corner of each panel, enlargement of one of the chromosomes with signals is shown in inset. Within a chromosome, a pale blue stained region corresponds to the centromeric region. The bar in **d** indicates 5 μm and is applicable to all panels. BAC clones used for probes were 5 J1 (Hox1), 3C14 (Hox2, Hox3 and Hox4), 6B23 (Hox5 and HoxX) and 1 J20 (Hox10, Hox11/12/13.a and Hox11/12/13.b)

In Fig. 3a, red and green spots corresponding to two BAC clones (one containing Hox1 and the other containing Hox2, Hox3 and Hox4, respectively) are shown located on the same chromosome. It is also noted here that Hox1 is localized closer to the chromosome end than Hox2, 3 and 4. In Fig. 3b, a green spot corresponding to the BAC clone containing Hox10, 11/12/13.a and Hox11/12/13.b was localized on the chromosome with a red spot representing Hox1. The red spot was closer to the chromosome end than the green spot (Fig. 3b). Figure 3c shows green and red spots corresponding to two BAC clones, one containing Hox5 and HoxX and the other containing the three posterior genes mentioned above, overlapping on a single chromosome. In Fig. 3d, Hox1 is localized closer to the chromosome end than Hox5, HoxX or the three posterior Hox genes. These results indicate that all of the nine Hox genes are present on a single chromosome in the *Hr* genome, unlike the *Ci* genome. In addition, these results suggest that eight of the nine Hox genes are localized closely together on the chromosome, although the positional relationship among the eight genes could not be determined in this analysis. In contrast, Hox1 was localized relatively close to the chromosome end, away from the other Hox genes. This arrangement is somewhat similar to that in the *Ci* genome (see Discussion), except that Hox12 and 13 genes are on a different chromosome in *Ci*.

### Hox gene order on the chromosome as inferred by chromosome/scaffold walking

Since chromosomal FISH analysis suggested that eight of nine Hox genes, excepting Hox1, may be localized in close proximity on a single chromosome, we examined overlapping of the BAC clones containing at least one of the eight Hox genes by reciprocal PCR using isolated BAC clone DNAs as templates. We found that many clones overlapped one another (data not shown), in a manner suggesting that Hox2, Hox3 and Hox4 form a subcluster and are aligned on the chromosome in this order. Similarly, five Hox genes, Hox5, HoxX, Hox10, Hox11/12/13.a and Hox11/12/13.b, form another subcluster and are aligned in the order, HoxX, Hox5, Hox10, Hox11/12/13.a, Hox11/12/13.b.

**Fig. 4:**
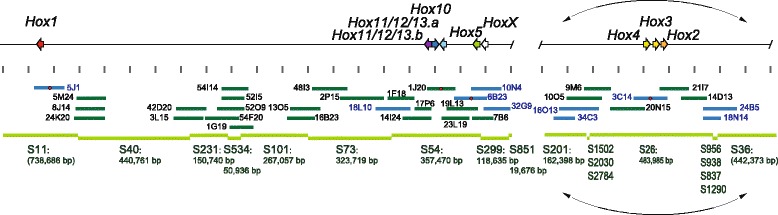
Schematic representation of the Hox gene cluster of *Halocynthia roretzi*. The Hox gene cluster structure of *Hr* as estimated by chromosome/scaffold walking is shown at the top. A horizontal line represents a part of chromosome. The telomeric side is to the left and the centromeric side is to the right. Hox genes are represented as thick arrows, which also indicate transcription direction. The color code is the same as that in Fig. 1. Between HoxX and Hox4, there is a region, from which no clones were available out of the BAC library; hence, no scaffolds available out of the ANISEED database. Grey arrays of short vertical bars indicate 100 kbp, starting at the right and left ends of the region where no scaffolds are available. Dark green or blue horizontal bars below the scales indicate BAC clones corresponding to Hox gene clusters. Dark green bars indicate BAC clones, end regions of which were sequenced. Blue bars indicate BAC clones for which the whole insert sequence was determined. Bars with red dots in the middle are the clones used for probes for chromosomal FISH (Fig. 3). Names of clones are also indicated. Green horizontal bars at the bottom indicate scaffolds in the ANISEED database (*Halocynthia roretzi* MTP2014; https://www.aniseed.cnrs.fr/fgb2/gbrowse/harore_mtp2014/) that correspond to the Hox gene cluster spanning chromosomal region. The length of each scaffold according to ANISEED database is indicated below scaffold names, except for the scaffolds in the two regions adjacent to S26, where multiple small scaffolds are included. Arc lines with arrowheads on both ends placed upper and lower of the subcluster region of Hox2, 3 and 4 indicate that the orientation of the subcluster has not been determined

In order to resolve the positional relationships between Hox1 and the two subclusters, we carried out chromosome/scaffold walking by utilizing the BAC library and genomic sequence information, *Halocynthia roretzi* MTP2014, in the genome browser of ANISEED database [20]. Scaffolds including Hox1 and the two subclusters were identified and the chromosome/scaffold walking was started from the ends of these scaffolds. We were successful in connecting the two scaffolds, S11 and S54, which contained Hox1 and Hox11/12/13.b, respectively (Fig. 4). At least five scaffolds were located between Hox1 and Hox11/12/13.b, and the distance between the two genes was about 1.53 Mbp according to calculations based on each scaffold length in the ANISEED database (Fig. 4). Walking distal to HoxX, no adjoining clone was isolated after isolation of a BAC clone (32G9 in Fig. 4), and the chromosome/scaffold walking was aborted. Similarly, walking distal to Hox4 was aborted, because no clone was isolated to connect scaffold S201 with its adjacent scaffold (Fig. 4). On the other hand, chromosome/scaffold walking distal to Hox2 yielded many clones at the end of scaffold S36 (Fig. 4). The clones contained similar, but not identical, nucleotide sequences at one end, and when used for BLAST queries to search the database, every clone hit many short scaffolds containing similar sequences. As a result, the scaffold neighboring S36 was not determined. These observations suggest that the Hox gene order on the chromosome is Hox1, Hox11/12/13.b, Hox11/12/13.a, Hox10, Hox5, HoxX, followed by either Hox4, Hox3, Hox2 or Hox 2, Hox3, Hox4, from the chromosome end to center (Fig. 4). In either case, the nine *Hr* Hox genes are estimated to span at least ~2.3 Mbp on the chromosome. A mir10 sequence that has been reported to reside in upstream of Hox4 in hemichordates and amphioxus [24–26] was not found in the *Hr* genome using BLAST survey over ANISEED genome browsers (data not shown).

## Discussion

Recent phylogenetic studies suggest that ascidians, comprising a major group of tunicates, are not monophyletic [27]. Tunicates are divided into two branches; one includes Stolidobranchia (*Hr*) and Appendicularia, and the other includes Phlebobranchia (*Ci*), Aplousobranchia (another ascidian group) and Thaliacea [27]. In the present study, we analyzed the Hox gene complement and the Hox gene cluster structure of the stolidobranchian ascidian, *Halocynthia roretzi*, which is phylogenetically remote from the phlebobranchian ascidian, *Ciona intestinalis*. The *Hr* Hox gene complement consists of nine members, the same as that of *Ci*. The nine *Hr* Hox genes are located on a single chromosome, unlike *Ci*, in which they reside on two chromosomes [11].

### The Hox gene complement in the last common ancestor of *Hr* and *Ci*

The present phylogenetic analysis suggested that eight of the nine *Hr* Hox gene complement are *Hr* orthologs of eight *Ci* Hox genes. When the remaining gene, *Hr* HoxX, was used to query various ascidian genomes in the ANISEED database, a Hox gene that exhibits high similarity was found only in the genome of a closely related species, *Halocynthia aurantium* (data not shown). On the other hand, in the ascidian species, *Ci* and *Ciona savignyi*, the best-hit Hox genes were *Ci-Hox6* and a probable *Cs* ortholog of *Ci-Hox6*, respectively (data not shown). In other ascidians, the best-hit gene was difficult to assign to a single PG during phylogenetic analysis (data not shown).

In the previous study, *Ci-Hox6* was tentatively designated as such, but without reliable evidence other than that it is localized proximal to *Ci-Hox5* (~3 kb apart, according to the ANISEED genome browser) [22]. In the present study, the phylogenetic position of the gene was close to, but not within the clade of PG6 genes, and it exhibited some affinity for PGs 7 and 8 (Fig. 1). The designation, *Ci-Hox6,* should thus be revisited in future studies.

Based on the above observations, we suggest that the last common ancestor to *Hr* and *Ci* may have possessed three central Hox genes, one of PG4, one of PG5 Hox genes, and one out of PGs 6–8 genes, and that the last one may have evolved in the lineages to *Hr* and *Ci*, and resulted in extant *Harore HoxX* and *Ci-Hox6*, respectively.

### The Hox gene complement of ascidians in comparison to that of amphioxus

The number of Hox genes in the *Hr* and *Ci* genomes is smaller than in amphioxus, which has 15 Hox genes [4, 5]. It seems reasonable that the ancestral tunicate may have lost several Hox genes after diverging from the evolutionary lineage leading to the vertebrates. However, the Hox genes of amphioxus are designated according to their order on the chromosome, not necessarily based on PGs, which were originally invented for classification of vertebrate Hox genes [2]. In recent years, studies have examined the relationship between amphioxus Hox genes and PGs, employing methods independent of phylogenetic tree construction [28, 29]. The results of these studies, although not necessarily concordant, suggest the common presence of each of PGs 1–5 genes and amphioxus-specific posterior gene paralogs in the genome [28, 29]. As regards the latter, it has been proposed that posterior Hox genes expanded in the amphioxus lineage, and that the last common ancestor of amphioxus and vertebrates may have possessed three ancestral posterior genes, PG9/10, PG11/12 and PG13/14 genes [30]. On the other hand, with respect to the anterior and central Hox genes, it is generally accepted that the last common ancestor of amphioxus and vertebrates possessed three anterior (PGs 1 through 3) and five central (PGs 4 through 8) Hox genes [30].

When amphioxus Hox protein sequences were analyzed in our phylogenetic tree (Additional file 1: Figure S1 and Additional file 2: Figure S2), amphioxus Hox1, 2, 3 and 4 were clearly classified into PGs1, 2, 3 and 4, respectively. Hox5 may be classified into PG5, although the clustering was not supported by a high bootstrap value. Amphioxus Hox6, 7, and 8 were grouped into a clade consisting of PGs 4–8 genes, but could be excluded from PG4 and PG5; thus, the three genes may be classified into PGs 6–8. Amphioxus Hox10, 11 and 12 apparently seemed to be paralogs (see Additional file 2: Figure S2) and were classified, together with Hox9, into a clade consisting of PGs 9 and 10, which was barely supported by a low bootstrap value. Similarly, amphioxus Hox13 and 14 seemed to be paralogs and were classified into PGs 11–13. By contrast, amphioxus Hox15 was apparently classified into PG13 with a relatively high bootstrap value (84%, Additional file 2: Figure S2).

Considering our observations and those of others, we speculate that the Hox gene cluster of the ancestral amphioxus (and the last common ancestor for amphioxus and vertebrates, too) comprised 11 Hox genes, including three anterior (each of PGs 1-3), five central (each of PGs 4–8), and three posterior (out of PGs 9–13) Hox genes. If this is the case, the last common ancestor of *Hr* and *Ci* must have lost two central Hox genes out of PGs 6–8 after divergence from the lineage continuing from the ancestral chordate to the ancestral vertebrate.

### Disintegration of the Hox gene cluster during evolution of *Hr* and *Ci*

In comparison with *Ci*, disintegration of the *Hr* Hox gene cluster seems less extensive, in that all nine Hox genes are on a single chromosome (Fig. 4). Thus, the Hox gene cluster of the last common ancestor of *Hr* and *Ci,* appears to have disintegrated differently in these two ascidians’ evolutionary lineages.

Nevertheless, there are some structural features shared by *Hr* and *Ci*. First, Hox1 is located away from other Hox genes in both *Hr* and *Ci* (Fig. 4, [11]). Second, Hox11/12/13.a and Hox11/12/13.b are adjacent to each other with reversed orientation. This situation is the same in *Ci* counterparts (*Ci-Hox12* and *Ci-Hox13*). Third, in both ascidians, Hox2, Hox3, and Hox4 are aligned in the same direction without intervening genes. It should be noted that in both ascidian genomes, the gene immediately adjacent to Hox2 is STAC (SH3 and cysteine-rich domain-containing protein) and two neighboring genes to Hox4 are CHST (carbohydrate sulfotransferase) and NEBL (Nebullet) (Additional file 3: Figure S3). This is the only conserved gene arrangement surrounding the Hox genes that we observed in these two ascidians. This suggests two possibilities. First, after divergence of the two lineages to *Hr* and *Ci,* genomic shuffling occurred in each lineage to such an extent that conservation of the gene arrangement surrounding the Hox genes was limited only to one small region, about 170 kb and 140 kb in *Hr* and *Ci*, respectively (see Additional file 3: Figure S3). Second, but more importantly, the gene arrangement observed in common between *Hr* and *Ci* must have been established prior to the divergence of *Hr* and *Ci*.

From these shared structural features*,* it appears that the disintegration of the Hox gene cluster must have included certain early events, such as translocation of Hox1 or of the Hox2, 3, 4 group, and tail-to-tail location of the Hox11/12/13.a and 11/12/13.b pair. These changes in the Hox gene cluster must have occurred in the last common ancestor of *Hr* and *Ci*.

## Conclusion: a theoretical scenario for the disintegration of the Hox gene cluster in the ascidian or tunicate evolution

**Fig. 5:**
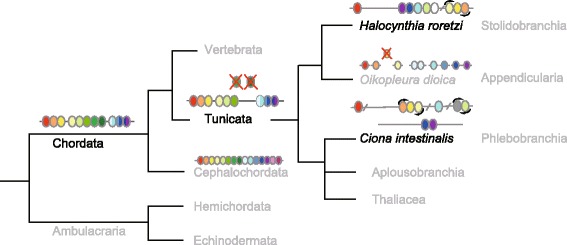
A proposed scheme for Hox gene cluster disintegration during ascidian evolution. The last common ancestor for cephalochordates, tunicates, and vertebrates (represented as Chordata) possessed a single Hox gene cluster consisting of three anterior (red, orange, and yellow), five central (green) and three ancestral posterior genes (blue). After the ancestral cephalochordate diverged, the tunicate ancestor (represented as Tunicata), in turn, diverged from the vertebrate lineage. At this stage, the ancestral tunicate must have experienced extensive changes in the genome, and the Hox gene cluster disintegration started, losing one or two central Hox genes. The ancestral tunicate subsequently evolved into two lineages, and in turn, diverged into Stolidobranchia and Appendicularia lineages (right side, upper) and Phlebobranchia, Aplousobranchia and Thaliacea lineages (right side, lower) [27]. The Hox gene complement of the ancestral tunicate with each three of anterior, central and posterior genes must have been established by the divergence of the two evolutionary lineages stated above. At the same time, early Hox gene cluster disintegration events must have occurred. In one of the two resultant evolutionary lineages, the ancestral stolidobranchial ascidian (*Hr*) emerged, being separated from the larvacean lineage. In the other evolutionary lineage, the ancestral phlebobranchial ascidian (*Ci*) emerged, being separated from Aplousobranchia and Thaliacea lineages. The Hox gene cluster subsequently disintegrated in different patterns in the two evolutionary lineages. White or gray ovals indicate Hox genes, probably of the central Hox gene group origin (see text). The Hox gene complement of *Oikopleura dioica*, consisting of two anterior, one central, and six posterior genes, and that of amphioxus, consisting of 15 members, are schematically represented

In an appendicularian tunicate, *Oikopleura dioica*, all central Hox genes and the PG3 Hox gene are missing, and the Hox gene complement in this species is quite different from that of *Hr* or *Ci* [31]. Considering this and information about Hox gene cluster of amphioxus, a simple scenario for the disintegration of the Hox gene cluster during the course of ascidian or tunicate evolution is as shown in Fig. 5.

In this scheme, 1) when the ancestral chordate emerged, it had a single Hox gene cluster consisting of three anterior (PGs 1–3), five central (PGs 4–8) and three ancestral posterior (PG9/10, PG11/12 and PG13/14) genes [30]. 2) The ancestral chordate evolved, and the last common ancestor of tunicates and vertebrates diverged from the lineage to cephalochordate. 3) When the ancestral tunicate diverged from the lineage to vertebrates, it must have experienced extensive genomic rearrangement and lost at least one (or two) central Hox genes. At the same time, the ancestral tunicate likely came to possess tunicate characteristics, and a Hox gene complement consisting of nine genes (three each of anterior, central, and posterior Hox genes) was established. Meanwhile, early disintegration events in the Hox gene cluster occurred. Loss of the central Hox genes and disintegration of the Hox gene cluster may be correlated with peculiar way of development of tunicates [12] and/or limited function of Hox genes as observed in the early development of *Ci* [32]. 4) The ancestral tunicate evolved and diverged into two distinct lineages, and in turn, ancestral ascidians of the Pleurogona (Stolidobranchia) and Enterogona (Phlebobranchia and Aplousobranchia) diverged from Appendicularia and Thaliacea, respectively. The Hox gene cluster as well as the genome must have experienced further genomic rearrangement. The relatively small conserved gene arrangement between *Hr* and *Ci* in the regions surrounding Hox genes may support this part of the scenario.

In the above simple scenario for the disintegration of the ascidian Hox gene cluster, it remains unresolved why one putative central Hox gene has diverged considerably more than other Hox genes in *Hr*. The evolutionary constraints governing the disintegration of the Hox gene cluster in *Hr* or *Ci*, which apparently occurred to a much smaller extent than in Appendicularia, also remain unknown. Answering these questions will further clarify the characteristic features of the Hox gene cluster in ascidians and/or tunicates.

## Additional files

Additional file 1: Figure S1. Amino acid sequences used for the analysis of Hox genes of *Halocynthia roretzi* (*Hr*) by construction of ML phylogenetic trees. Amino acid sequences include the homeodomain (60 residues in yellow) and the adjacent 20 N-terminal and seven C-terminal residues. Original accession numbers for these sequences are indicated in brackets. Taxonomic abbreviations are Mm for *Mus musculus*, Lm for *Latimeria menadoensis*, Hf for *Heterodontus francisci*, Ci for *Ciona intestinalis*, Hr for *Halocynthia roretzi*, Bl for *Branchiostoma lanceolatum* and Bf for *Branchiostoma floridae*. *Hr* Hox genes, designated according to orthology with *Ci-Hox* counterparts and according to their classification into paralog groups (PGs) are indicated prior to and in parentheses, respectively. In ascidian sequences, letters in red indicate diagnostic residues for Hox10 homeodomain proteins (see text). (PDF 41 kb)

Additional file 2: Figure S2. Phylogenetic analysis of amphioxus Hox genes by constructing an ML tree. The ML tree was constructed using homeodomain sequences and the adjacent 20 N-terminal and seven C-terminal amino acids (Additional file 1: Figure S1) and MEGA5 software. The percentage of 1000 replicated trees, in which gene clustering was supported, is indicated at nodes. Within a clade consisting of only vertebrate Hox genes, the percentage was not indicated at the node. Amphioxus Hox genes are marked by colored circles. Color code and taxonomic abbreviations are the same as in Fig. 1, except that the color code for posterior Hox genes is the same as that shown in Fig. 5. Bl and Bf denote *Branchiostoma lanceolatum* and *Branchiostoma floridae*, respectively. (PDF 519 kb)

Additional file 3: Figure S3. Conservation of gene arrangements surrounding Hox2, 3, and 4 between *Hr* and *Ci*. Genomic regions surrounding Hox2, Hox3, and Hox4 are depicted schematically, based on genomic browser information from the ANISEED database (*Halocynthia roretzi* MTP 2014, *Ciona intestinalis* type A (KH2012)). Genes are indicated by thick arrows. The color code for Hox genes is the same as in Fig. 4. Pink arrows downstream of Hox2 indicate the gene encoding SH3 and cysteine-rich domain-containing protein (STAC). Pale pink arrows upstream of Hox4 indicate carbohydrate sulfotransferase (CHST1)/chondroitin 6-O-sulfotransferase (C6ST). Dark pink arrows indicate the NEBL gene encoding the Nebullete protein. Blank arrows indicate genes without positional conservation. Grey arrays of short vertical bars indicate 10 kbp. (PDF 246 kb)
