# Supplementary material for: Hox gene cluster of the ascidian, Halocynthia roretzi, reveals multiple ancient steps of cluster disintegration during ascidian evolution
Source: Zoological Lett. 2017 Sep 15;3:17. doi: 10.1186/s40851-017-0078-3 (PMC5602962; doi:10.1186/s40851-017-0078-3)
Supplement: Supplementary file 1 — Amino acid sequences used for the analysis of Hox genes of Halocynthia roretzi (Hr) by construction of ML phylogenetic trees. Amino acid sequences include the homeodomain (60 residues in yellow) and the adjacent 20 N-terminal and seven C-terminal residues. Original accession numbers for these sequences are indicated in brackets. Taxonomic abbreviations are Mm for Mus musculus, Lm for Latimeria menadoensis, Hf for Heterodontus francisci, Ci for Ciona intestinalis, Hr for Halocynthia roretzi, Bl for Branchiostoma lanceolatum and Bf for Branchiostoma floridae. Hr Hox genes, designated according to orthology with Ci-Hox counterparts and according to their classification into paralog groups (PGs) are indicated prior to and in parentheses, respectively. In ascidian sequences, letters in red indicate diagnostic residues for Hox10 homeodomain proteins (see text). (PDF 41 kb) [file 40851_2017_78_MOESM1_ESM.pdf]

Mm_Hox-A1[NP_034579.3]
KVKRNPPKTGKVGEYGYVGQPNAVRTNFTTKQLTELEKEFHFNKYLTRARRVEIAASLQLNETQVKIWFQNRRMKQKKREKEGLLPI

Mm_Hox-B1[NP_032292.3]
MKVKRNPPKTAKVSELGLGAPGGLRTNFTTRQLTELEKEFHFNKYLSRARRVEIAATLELNETQVKIWFQNRRMKQKKREREGGRMP

Mm_Hox-D1[NP_034597.2]
KVKRNAPKKSKLSEYGATSPPSAIRTNFSTKQLTELEKEFHFNKYLTRARRIEIANCLQLNDTQVKIWFQNRRMKQKKREREGLLAT

Mm_Hox-A2[NP_034581.1]
GPACLGHKESLEIADGSGGGSRRLRTAYTNTQLLELEKEFHFNKYLCRPRRVEIAALLDLTERQVKVWFQNRRMKHKRQTQCKENQN

Mm_Hox-B2[NP_598793.2]
ASEVGSPSDGPGLPECGGSGSRRLRTAYTNTQLLELEKEFHFNKYLCRPRRVEIAALLDLTERQVKVWFQNRRMKHKRQTQHREPPE

Mm_Hox-A3[NP_034582.1]
SGSSSGESCAGDKSPPGQASSKRARTAYTSAQLVELEKEFHFNRYLCRPRRVEMANLLNLTERQIKIWFQNRRMKYKKDQKGKGMLT

Mm_Hox-B3[NP_034588.2]
SGGGGGGGGGGDKSPPGSAASKRARTAYTSAQLVELEKEFHFNRYLCRPRRVEMANLLNLSERQIKIWFQNRRMKYKKDQKAKGLAS

Mm_Hox-D3[NP_034598.2]
KNSCATSGENCEDKSPPGPASKRVRTAYTSAQLVELEKEFHFNRYLCRPRRVEMANLLNLTERQIKIWFQNRRMKYKKDQKAKGILH

Mm_Hox-A4[NP_032291.1]
YPWMKKIHVSAVNSSYNGGEPKRSRTAYTRQQVLELEKEFHFNRYLTRRRRIEIAHTLCLSERQVKIWFQNRRMKWKKDHKLPNTKM

Mm_Hox-B4[NP_034589.3]
YPWMRKVHVSTVNPNYAGGEPKRSRTAYTRQQVLELEKEFHYNRYLTRRRRVEIAHALCLSERQIKIWFQNRRMKWKKDHKLPNTKI

Mm_Hox-C4[NP_038581.2]
YPWMKKIHVSTVNPNYNGGEPKRSRTAYTRQQVLELEKEFHYNRYLTRRRRIEIAHSLCLSERQIKIWFQNRRMKWKKDHRLPNTKV

Mm_Hox-D4[NP_034599.2]
YPWMKKVHVNSVNPNYTGGEPKRSRTAYTRQQVLELEKEFHFNRYLTRRRRIEIAHTLCLSERQIKIWFQNRRMKWKKDHKLPNTKG

Mm_Hox-A5[NP_034583.1]
QIYPWMRKLHISHDNIGGPEGKRARTAYTRYQTLELEKEFHFNRYLTRRRRIEIAHALCLSERQIKIWFQNRRMKWKKDNKLKSMSM

Mm_Hox-B5[NP_032294.2]
PQIFPWMRKLHISHDMTGPDGKRARTAYTRYQTLELEKEFHFNRYLTRRRRIEIAHALCLSERQIKIWFQNRRMKWKKDNKLKSMSL

Mm_Hox-C5[NP_783857.1]
PAPPQIYPWMTKLHMSHETDGKRSRTSYTRYQTLELEKEFHFNRYLTRRRRIEIANNLCLNERQIKIWFQNRRMKWKKDSKMKSKEA

Mm_Hox-A6[NP_034584.1]
PVYPWMQRMNSCAGAVYGSHGRRGRQTYTRYQTLELEKEFHFNRYLTRRRRIEIANALCLTERQIKIWFQNRRMKWKKENKLINSTQ

Mm_Hox-B6[NP_032295.1]
PVYPWMQRMNSCNSSSFGPSGRRGRQTYTRYQTLELEKEFHYNRYLTRRRRIEIAHALCLTERQIKIWFQNRRMKWKKESKLLSASQ

Mm_Hox-C6[NP_034595.2]
QIYPWMQRMNSHSGVGYGADRRRGRQIYSRYQTLELEKEFHFNRYLTRRRRIEIANALCLTERQIKIWFQNRRMKWKKESNLTSTLS

Mm_Hox-A7[NP_034585.1]
HGPAEASFRIYPWMRSSGPDRKRGRQTYTRYQTLELEKEFHFNRYLTRRRRIEIAHALCLTERQIKIWFQNRRMKWKKEHKDESQAP

Mm_Hox-B7[NP_034590.2]
DLAAESNFRIYPWMRSSGPDRKRGRQTYTRYQTLELEKEFHYNRYLTRRRRIEIAHTLCLTERQIKIWFQNRRMKWKKENKTSGPGT

Mm_Hox-B8[NP_034591.1]
EQSPSPTQLFPWMRPQAAAGRRRGRQTYSRYQTLELEKEFLFNPYLTRKRRIEVSHALGLTERQVKIWFQNRRMKWKKENNKDKFPS

Mm_Hox-C8[NP_034596.1]
LNQNSSPSLMFPWMRPHAPGRRSGRQTYSRYQTLELEKEFLFNPYLTRKRRIEVSHALGLTERQVKIWFQNRRMKWKKENNKDKLPG

Mm_Hox-D8[NP_032302.2]
NQSSSPSQMFPWMRPQAAPGRRRGRQTYSRFQTLELEKEFLFNPYLTRKRRIEVSHTLALTERQVKIWFQNRRMKWKKENNKDKFPA

Mm_Hox-A9[NP_034586.1]
GDKPPIDPNNPAANWLHARSTRKKRCPYTKHQTLELEKEFLFNMYLTRDRRYEVARLLNLTERQVKIWFQNRRMKMKKINKDRAKDE

Mm_Hox-B9[NP_032296.2]
EDKERPDQTNPSANWLHARSSRKKRCPYTKYQTLELEKEFLFNMYLTRDRRHEVARLLNLSERQVKIWFQNRRMKMKKMNKEQGKE*

Mm_Hox-C9[NP_032298.1]
EEKADLDPSNPVANWIHARSTRKKRCPYTKYQTLELEKEFLFNMYLTRDRRYEVARVLNLTERQVKIWFQNRRMKMKKMNKEKTDKE

Mm_Hox-D9[NP_038583.1]
PPQQQLDPNNPAANWIHARSTRKKRCPYTKYQTLELEKEFLFNMYLTRDRRYEVARILNLTERQVKIWFQNRRMKMKKMSKEKCPKG

Mm_Hox-A10[NP_032289.2]
KDSLGSSKGENAANWLTAKSGRKKRCPYTKHQTLELEKEFLFNMYLTRERRLEISRSVHLTDRQVKIWFQNRRMKLKKMNRENRIRE

Mm_Hox-C10[NP_034592.2]
EAKEEIKAENTTGNWLTAKSGRKKRCPYTKHQTLELEKEFLFNMYLTRERRLEISKTINLTDRQVKIWFQNRRMKLKKMNRENRIRE

Mm_Hox-D10[NP_038582.2]
ESKEEIKSDTPTSNWLTAKSGRKKRCPYTKHQTLELEKEFLFNMYLTRERRLEISKSVNLTDRQVKIWFQNRRMKLKKMSRENRIRE

Mm_Hox-A11[P31311.2]
SPESSSGHTEDKAGGSGGQRTRKKRCPYTKYQIRELEREFFFSVYINKEKRLQLSRMLNLTDRQVKIWFQNRRMKEKKINRDRLQYY

Mm_Hox-C11[NP_001020013.1]
GSSHSATKEPAKGAAPNAPRTRKKRCPYSKFQIRELEREFFFNVYINKEKRLQLSRMLNLTDRQVKIWFQNRRMKEKKLSRDRLQYF

Mm_Hox-D11[NP_032299.1]
EGPPGEAGAEKSGGTVAPQRSRKKRCPYTKYQIRELEREFFFNVYINKEKRLQLSRMLNLTDRQVKIWFQNRRMKEKKLNRDRLQYF

Mm_Hox-C12[NP_034593.1]
LNPGGGLSASGAPWYPIHSRSRKKRKPYSKLQLAELEGEFLVNEFITRQRRRELSDRLNLSDQQVKIWFQNRRMKKKRLLLREQALS

Mm_Hox-D12[NP_032300.2]
ASCLRSSLPDGLPWGAAPGRARKKRKPYTKQQIAELENEFLVNEFINRQKRKELSNRLNLSDQQVKIWFQNRRMKKKRVVQREQALA

Mm_Hox-A13[NP_032290.1]
WKSTLPDVVSHPSDASSYRRGRKKRVPYTKVQLKELEREYATNKFITKDKRRRISATTNLSERQVTIWFQNRRVKEKKVINKLKTTS

Mm_Hox-B13[AAH51087.2]
AAFAEPSVQHPPPDGCAFRRGRKKRIPYSKGQLRELEREYAANKFITKDKRRKISAATSLSERQITIWFQNRRVKEKKVLAKVKTST

Mm_Hox-C13[NP_034594.1]
WKSPFPDVVPLQPEVSSYRRGRKKRVPYTKVQLKELEKEYAASKFITKEKRRRISATTNLSERQVTIWFQNRRVKEKKVVSKSKAPH

Mm_Hox-D13[NP_032301.2]
KSSFPGDVALNQPDMCVYRRGRKKRVPYTKLQLKELENEYAINKFINKDKRRRISAATNLSERQVTIWFQNRRVKDKKIVSKLKDTV

Lm_HoxA1[ACL81429.1]
KVKRNPPKTGKVGEYGYAGQPNTVRTNFTTKQLTELEKEFHFNKYLTRARRVEIAAALQLNETQVKIWFQNRRMKQKKREKEGLIPV
Lm_HoxB1[ACL81442.1]
KVKRNPPKTAKVAEYGVNGQQNTIRTNFTTKQLTELEKEFHFNKYLTRARRVEIAATLELNETQVKIWFQNRRMKQKKREREGITST
Lm_HoxC1[ACL81453.1]
MKVKRNPPRTGDCVYSGESNGATARTNFTTKQLTELEKEFHFNKYLTRARRVEIASALQLNETQVKIWFQNRRMKQKKREREGLAVG
Lm_HoxD1[ACL81464.1]
KVKRNPPKTSKPTEFGVCSPVNTARTNFTTKQLTELEKEFHFNKYLSRTRRIEIANALHLNETQVKIWFQNRRMKQKKRGQVGLLST
Lm_HoxA2[ACL81434.1]
GPACFTQKESPEIPDTAGGGSRRLRTAYTNTQLLELEKEFHFNKYLCRPRRVEIAALLDLTERQVKVWFQNRRMKHKRQTQCKENQN
Lm_HoxB2[ACL81445.1]
SGSGVGSPTEAQGLQDNSNGSRRLRTAYTNTQLLELEKEFHFNKYLCRPRRVEIAALLDLTERQVKVWFQNRRMKHKRQTQHKDNHE
Lm_HoxB3[ACL81446.1]
NSSPSTESCSGDKSPPGSSASKRARTAYTSAQLVELEKEFHFNRYLCRPRRVEMANLLNLSERQIKIWFQNRRMKYKKDQKSKGMGS
Lm_HoxA3[ACL81435.1]
SSCSSGDGCTGDKSPPGPASSKRARTAYTSAQLVELEKEFHFNRYLCRPRRVEMANLLNLTERQIKIWFQNRRMKYKKDQKGKGMMT
Lm_HoxD3[ACL81468.1]
KNNCTATGESCEEKSPSGPTSKRVRTAYTSAQLVELEKEFHFNRYLCRPRRVEMANLLNLTERQIKIWFQNRRMKYKKDQKAKGIMH
Lm_HoxA4[ACL81436.1]
YPWMKKIHVCTVNPNYNGGEPKRSRTAYTRQQVLELEKEFHFNRYLTRRRRIEIAHTLCLSERQVKIWFQNRRMKWKKDHKLPNTKM
Lm_HoxB4[ACL81447.1]
PIVYPWMKKVHVNPNFTGGEPKRSRTAYTRQQVLELEKEFHYNRYLTRRRRVEIAHSLCLSERQIKIWFQNRRMKWKKDHKLPNTKI
Lm_HoxC4[ACL81459.1]
YPWMKKIHVSTVNPNYNGGEPKRSRTAYTRQQVLELEKEFHYNRYLTRRRRIEIAHSLCLSERQIKIWFQNRRMKWKKDHRLPNTKV
Lm_HoxD4[ACL81469.1]
YPWMKKVHVNSVNPNYNGGEPKRSRTAYTRQQVLELEKEFHFNRYLTRRRRIEIAHTLCLSERQIKIWFQNRRMKWKKDHKLPNTKG
Lm_HoxB5[ACL81448.1]
PQIFPWMRKLHISHDMTGPDGKRARTAYTRYQTLELEKEFHFNRYLTRRRRIEIAHALCLSERQIKIWFQNRRMKWKKDNKLKSMSL
Lm_HoxA5[ACL81437.1]
QIYPWMRKLHISHDSIGGPEGKRARTAYTRYQTLELEKEFHFNRYLTRRRRIEIAHALCLSERQIKIWFQNRRMKWKKDNKLKSMSM
Lm_HoxC5[ACL81460.1]
QSQPQIYPWMTKLHMSHDTDGKRSRTSYTRYQTLELEKEFHFNRYLTRRRRIEIANNLCLNERQIKIWFQNRRMKWKKDSKLKSKEP
Lm_HoxA6[ACL81438.1]
PIFPWMQRMNSCTGTEYGTHGRRGRQTYTRYQTLELEKEFHFNRYLTRRRRIEIANALCLTERQIKIWFQNRRMKWKKESKLLNSTQ
Lm_HoxB6[ACL81449.1]
PVYPWMQRMNSCTGSAFGPNGRRGRQTYTRYQTLELEKEFHFNRYLTRRRRIEIAHALCLTERQIKIWFQNRRMKWKKENKLLGASQ
Lm_HoxA7[ACL81439.1]
HPQDENNFRIYPWMRSSGPDKKRGRQTYTRYQTLELEKEFHFNRYLTRRRRIEIAHALCLTERQIKIWFQNRRMKWKKEHKEDNFTS
Lm_HoxB7[ACL81450.1]
EQQNESNFRIYPWMRSTGPDRKRGRQTYTRYQTLELEKEFHYNRYLTRRRRIEIAHALCLTERQIKIWFQNRRMKWKKENKTTTQSL
Lm_HoxC8[ACL81462.1]
LNQNSSPSLMFPWMRPHAPGRRSGRQTYSRYQTLELEKEFLFNPYLTRKRRIEVSHALGLTERQVKIWFQNRRMKWKKENNKDKLPG
Lm_HoxD8[ACL81470.1]
QETSSPSQMFPWMRSQAATGRRRGRQTYSRFQTLELEKEFLFNPYLTRKRRIEVSHSLGLTERQIKIWFQNRRMKWKKENNKDTFST
Lm_HoxA9[ACL81440.1]
GDKPQIDPNNPAANWLHARSTRKKRCPYTKHQTLELEKEFLFNMYLTRDRRYEVARLLNLTERQVKIWFQNRRMKMKKINKDRSKDE
Lm_HoxC9[ACL81463.1]
EEKAELDPNNPVANWIHARSTRKKRCPYTKYQTLELEKEFLFNMYLTRDRRYEVARVLNLTERQVKIWFQNRRMKMKKMNKEKNDKE
Lm_HoxD9[ACL81471.1]
EKPKQLDPNNPAAIWLHARSTRKKRCPYTKYQTLELEKEFLFNMYLTRDRRYEVARILDLTERQVKIWFQNRRMKMKKMNKERGNKE
Lm_HoxA10[ACL81430.1]
KEAIGNAKGENAANWLTAKSGRKKRCPYTKHQTLELEKEFLFNMYLTRERRLEISRSVHLTDRQVKIWFQNRRMKLKKMNRENRIRE
Lm_HoxC10[ACL81454.1]
EAKEDMKTESATGNWLTAKSGRKKRCPYTKHQTLELEKEFLFNMYLTRERRLEISKSINLTDRQVKIWFQNRRMKLKKMNRENRIRE
Lm_HoxD10[ACL81465.1]
EGKDGGKSEVSTSNWLTAKSGRKKRCPYTKHQTLELEKEFLFNMYLTRERRLEISRSVNLTDRQVKIWFQNRRMKLKKMSRENRIRE
Lm_HoxA11[ACL81431.1]
SPESSSGNNEEKSSSSSGQRTRKKRCPYTKYQIRELEREFFFSVYINKEKRLQLSRMLNLTDRQVKIWFQNRRMKEKKLNRDRLQYY
Lm_HoxD11[ACL81466.1]
TDLPSDKVVAEKHSNSTSVRSRKKRCPYSKFQIRELEREFFFNVYINKEKRLQLSRMLNLTDRQVKIWFQNRRMKEKKLNRDRLQYF
Lm_HoxC12[ACL81456.1]
ISNGNSLSTAGAPWYPMHTRSRKKRKPYSKLQLAELEGEFMVNEFITRQRRELSDRLNLSDQQVKIWFQNRRMKKKRLLLREQALS
Lm_HoxD12[ACL81467.1]
QSCTRPALTEGLTWCPTQVRSRKKRKPYTKQQIAHLENEFLINEFINRQKRKELSDRLNLSDQQVKIWFQNRRMKKKRLIMREQTLS
Lm_HoxA13[ACL81432.1]
WKSTLPDVVSHPSDANSFRRGRKKRVPYTKVQLKELEREYATNKFITKDKRRRISATTNLSERQVTIWFQNRRVKEKKVINKLKTTS
Lm_HoxB13[ACL81444.1]
WKSALADVVAHQQDGSSFRRGRKKRIPYTKVQLKELEKEYATNKFITKDKRRKISAATNLSERQITIWFQNRRVKEKKVVAKIKPTT
Lm_HoxC13[ACL81457.1]
WKSPFPDVVPLQPEVNSYRRGRKKRVPYTKIQLKELEKEYAASKFITKEKRRRISATTNLSERQVTIWFQNRRVKEKKILSKSKAAH

HF_HOXA1[AAF44639.1]
KVKRNPPKTGKAGEYGFAGQPNTVRTNFTTKQLTELEKEFHFNKYLTRARRVEIAAALQLNETQVKIWFQNRRMKQKKREKEGLTSA
HF_HOXD1[ACY39980.1]
KVKRNPPKTARTTDYGVPSPTSTARTNFTTKQLTELEKEFHFNKYLTRARRVEIANALQLSETQVKIWFQNRRMKQKKRERDGFLAN
HF_HOXA2[AAF44640.1]
AASCLSQKETHEIPDNTGGGSRRLRTAYTNTQLLELEKEFHFNKYLCRPRRVEIAALLDLTERQVKVWFQNRRMKHKRQTQCKENQN
HF_HOXD2[ACY39979.1]
LLATENEDLNEPDNGSPFDSSRRLRTTYTNTQLLELEKEFHYNRYLCRPRRVEIAALLDLTERQVKVWFQNRRMKHKRQTRFKQSQN
HF_HOXA3[AAF44641.1]
TSSSSSVESSAGEKSPPGPASKRARTAYTSAQLVELEKEFHFNRYLCRPRRVEMANLLNLTERQIKIWFQNRRMKYKKDQKAKGMLT
HF_HOXD3[ACY39978.1]
KNNCTVAGDNCEDKSPPGPSSKRVRTAYTSAQLVELEKEFHFNRYLCRPRRVEMANLLNLTERQIKIWFQNRRMKYKKDQKSKGIMH
HF_HOXA4[AAF44642.1]
YPWMKKIHVTTVNPNYTGGEPKRSRTAYTRQQVLELEKEFHFNRYLTRRRRIEIAHTLCLSERQVKIWFQNRRMKWKKDHKLPNTKM
HF_HOXD4[ACY39977.1]
YPWMKKIHVNTVNPNYTGGEPKRSRTAYTRQQVLELEKEFHFNRYLTRRRRIEIAHTLCLSERQIKIWFQNRRMKWKKDHKLPNTKT
HF_HOXA5[AAF44643.1]
QIYPWMRKLHISHDSMGGPEGKRARTAYTRYQTLELEKEFHFNRYLTRRRRIEIAHALCLTERQIKIWFQNRRMKWKKDNKLKSMSM
HF_HOXD5[AAF44631.1]
QIYPWMKKMHLNQEGLSGLEGKRTRTAYTRYQTLELEKEFHYNRYLTRRRRIEIAHALCLTERQIKIWFQNRRMKWKKDNKLKSINV
HF_HOXA6[AAF44644.1]
PIYPWMQRMNSSSSSVFGPHGRRGRQTYTRFQTLELEKEFHFNRYLTRRRRIEIANALCLTERQIKIWFQNRRMKWKKENKLLNTTE
HF_HOXA7[AAF44645.1]
HPQAESNFRIYPWMRNAGPDRKRGRQTYTRYQTLELEKEFHFNRYLTRRRRIEIAHALCLTERQIKIWFQNRRMKWKKETKAGSSST
HF_HOXD8[AAF44632.1]
NQNSSPTQMFPWMRPQAAPGRRRGRQTYSRFQTLELEKEFLFNPYLTRKRRIEVSHALGLTERQVKIWFQNRRMKWKKENNKDKFPT
HF_HOXA9[AAF44646.1]
ADKLHMDPNNPSANWLHARSTRKKRCPYTKHQTLELEKEFLFNMYLTRDRRYEVARVLNLTERQVKIWFQNRRMKMKKINKERPKDD
HF_HOXD9[AAF44633.1]
EKQQQLDPNHPAINWIHARSTRKKRCPYTKYQTLELEKEFLFNMYLTRDRRYEVARILNLTERQVKIWFQNRRMKMKKMNKVKNNEE
HF_HOXA10[AAF44647.1]
KETKGEVKAENGANWLTAKSGRKKRCPYTKHQTLELEKEFLFNMYLTRERRLEISRSVHLTDRQVKIWFQNRRMKLKKMNRENRIRE
HF_HOXD10[AAF44634.1]
DNETKEEIKTPISNWLTAKSGRKKRCPYTKYQTLELEKEFLFNMYLTRERRLEISKSVNLTDRQVKIWFQNRRMKLKKMNRETRIRE
HF_HOXD11[AAF44635.1]
TVESSGTSATEKNSLSTALRCRKKRCPYTKYQIRELEREFFFNVYINKEKRLQLSRMLNLTDRQVKIWFQNRRMKEKKLSRDRLHFF
HF_HOXD12[AAF44636.1]
VTPVCNRSSDGLPWCPTQVRSRRKRKPYTKQQIAELENEFLANEFINRQKRKELSDRLNLSDQQVKIWFQNRRMKKKRLVMREQTLS
Hf_HoxD13[AAF44637.1]
KSPLSGDVMHNQTDINIYRRGRKKRVPYTKTQLKELEREYATNKFITKEKRRRISTATNLTERQVTIWFQNRRVKEKKVVSKVKENI

Ci-Hox1[NP_001122333.1]
PPKSIYFSQNKMVEYTYGVTGNNGRTNFTTKQLTELEKEFHFNKYLTRARRVEIAAALRLNETQVKIWFQNRRMKQKKRDKEAEKLN
Ci-Hox2[CAD59668.1]
--------------VRPAGASRRLRTAYTNTQLLELEKEFHYNKYLCRPRRIEIATLLDLTERQVKVWFQNRRMKHKRQQLQQSQKD
Ci-Hox3[NP_001027669.1]
KHSSSESNSSSGDGDKCYSASKRERTAYTNSQLVELEKEFHFSHYLCRPRRIELAQGLGLTERQIKIWFQNRRMKFKKEQKQKAVLQ
Ci-Hox4[NP_001027781.2]
VVYPWMKRIHVSQVINGLESGKRPRTAYTRHQVLELEKEFHYNRYLTRRRRIEIAHGLCLSERQVKIWFQNRRMKWKKDHKLPNTKV
Ci-Hox5[CAA05151.1]
RDAIIYPWMKRIHGGETPDPSKRTRTAYTRYQTLELEKEFHYNRYLTRRRRIEVAHTLCLTERQIKIWFQNRRMKWKKENKLSSLNS
Ci-Hox6[CAD59670.1]
SIYCDIYITSFKQPISTGSHRRRGRQTYSRHQTLELEKEFHYNRYLTRRRRIEVAHTLCLTERQIKIWFQNRRMKWKKENKDIAESN
Ci-Hox10[NP_001027696.1]
EETEDAGEEADPTKHWLTASGRKKRVPYTKYQLLELEKEFHYNQYLSRERRLEVAKSVKLTDRQVKIWFQNRRMKWKKERREERQRD
Ci-Hox12[NP_001071736.1]
PYQTVLPGQHYPVDVTRHGAQRRRRRPYTKYQLSELEREFGANEFISREMREQIAVRVGLNDRQVKIWFQNRRMKKKRMQHRGEQSV
Ci-Hox13[NP_001122344.1]
VMTVSAYCCAALGLQHGPSHSRKKRQPYSKTQISSLEREYKANNFITRQKRENIARDLKLSDRQVKIWFQNRRVKDKKIKQREIKDN

Mm_Nkx-2.1[NP_033411.3]
GGLGSLGDVSKNMAPLPSAPRRKRRVLFSQAQVYELERRFKQQKYLSAPEREHLASMIHLTPTQVKIWFQNHRYKMKRQAKDKAAQQ
Lc_Nkx-2.1[XP_005989231.1]
GSLGSLGEVGKSMAPLQSTPRRKRRVLFSQAQVYELERRFKQQKYLSAPEREHLASMIHLTPTQVKIWFQNHRYKMKRQAKDKAAQQ
Bf_Nkx-2.1[AAC35350.1]
NMGMSLGTIEGPKPILPTTQRRKRRVLFSQAQVYELERRFKQQKYLSAPEREHLAQLINLTPTQVKIWFQNHRYKCKRQDKERQKSS

```
HrHox1(Harore_Hox1[LC272074])
  PKTNGFYTHGKMDAYGYAGTAGNGRTNFTTKQLTELEKEFHFNKYLTRARRVEIAAALLLNETQVKIWFQNRRMKQKKRDKEAEKLI
HrHox2(Harore_Hox2[LC272075])
  QLLPLTENMNFDTGIRPPGTSRRLRTAYTNTQLLELEKEFHYNKYLCRPRRIEIATLLDLTERQVKVWFQNRRMKHKRQQQQSKQDG
HrHox3(Harore_Hox3[LC272076])
  QNARRKQGSSSSMSSNGDKSSKRERTAYTNSQLVELEKEFHFNRYLCRPRRIEMAQMLSLTERQIKIWFQNRRMKYKKDQKLKVMPI
HrHox4(Harore_Hox4[LC272077])
  VYPWMKRIHVSHVFNGSIEPCKRPRTAYTRHQVLELEKEFHFNRYLTRRRRIEIAHSLCLTERQVKIWFQNRRMKWKKDNKLPNTKS
HrHox5(Harore_Hox5[LC272078])
  EYWYRQHIKLILFLDEALDSVKRTRTAYTRYQTLELEKEFHFNRYLTRRRRIEIAHVLCLTERQIKIWFQNRRMKWKKENKIKSLNS
HrHoxX(Harore_HoxX[LC272079])
  ANFPNVCLIPMLTYSFPDIPSQRPRQIYSRHQTLELEKEFHFCKYVTYKDRMKIANTLNLTERQVKIWFQNRRMKSKKVERKATAAI
HrHox10(Harore_Hox10[LC272080])
  DDKLGGGKGEDPTKHWLTASGRKKRVPYTKYQLLELEKEFHYNQYLSRERRLEVAKTVNLTDRQVKIWFQNRRMKWKKEKKEEKIRD
HrHox12(Harore_Hox11/12/13.a[LC272081])
  YTTIGNNLSPFHPGHMHPASSRKRRKPYTKYQLQELEKEFNANEFISRELRLQIAKRVFLSDRQVKIWFQNRRMKKKRMHQREKNGD
HrHox13(Harore_Hox11/12/13.b[LC272082])
  PPTYSNAMHHHLAHSHHSSSSRKKRQPYSKSQIAHLEKEYKKNNFITRQKREQISKELSLTDRQVKIWFQNRRVKDKKMKQRETREA


Bl_Hox1[AFO68803.1]
  LKRNPPRTGKPGEYGFTTSGPNNGRTNFTTKQLTELEKEFHYNKYLTRARRVEIAAALNLNETQVKIWFQNRRMKQKKREKENGFST
Bl_Hox2[AFV93978.1]
  PVFNTQEADAFNSPTDRESSSRRLRTVFTNTQLLELEKEFHYNKYVCKPRRKEIASFLDLNERQVKIWFQNRRMRQKRRDTKSRSEI
Bl_Hox3[AFO68805.1]
  TNLSVGTTETGESPGLGGAAGKRARTAYTSAQLVELEKEFHFNRYLCRPRRVEMAAMLNLTERQIKIWFQNRRMKYKKEQKVKGGGS
Bl_Hox4[AFO68807.1]
  YPWMKKVHSNTGSTSYNGQDPKRSRTAYTRQQVLELEKEFHFNRYLTRRRRIEIAHSLGLTERQIKIWFQNRRMKWKKDNRLPNTKT
Bl_Hox5[AFV93980.1]
  IPMYPWMRKIHLNHSAGTGDNKRTRTAYTRYQTLELEKEFHFNRYLTRRRRIEIAHALCLTERQIKIWFQNRRMKWKKENKLKSLSQ
Bl_Hox6[AFO68809.1]
  TPPVFPWMRKGSSQTAMGEEKKRGRQTYTRYQTLELEKEFHFNKYLTRKRRIEIAHLLGLTERQIKIWFQNRRMKWKKENKIPSLNA
Bl_Hox7[AFO68811.1]
  LNTAQMTTPIYPWMRSTAPERKRGRQTYTRYQTLELEKEFHFNKYLTRRRRIEIAHALCLTERQIKIWFQNRRMKWKKENKLESLKQ
Bl_Hox8[AFV93982.1]
  AQQSQLAIPFYPWMRTAGPERRRGRQTYSRYQTLELEKEFHFNRYLTRRRRIEIAHALGLTERQIKIWFQNRRMKLKKEAAMLCPPK
Bl_Hox9[ACJ74386.1]
  GDDKHGSALANQPGWMNNHSSRKKRCPYTRFQTLELEKEFLYNMYLTRERRYEISQHVNLSERQVKIWFQNRRMKMKKMSKQRQEQQ
Bl_Hox10[ACJ74389.1]
  TNGASPLHVCGATSWMAPRVGRKKRCPYTKYQILELEKEFLFNMYVSRERRQEISRHVNLSDRQVKIWFQNRRMKMKRMNKAREEQI
Bf_Hox11[AAF81909.1]
  -----------TSNWMSAKSTRKKRCPYTKYQTLELEKEFLFNMFVTRERRQEIARQLNLTDRQVKIWFQNRRMKMKRMKQRAMQQL
Bl_Hox12[AAF81909.1]
  TADGSEADPNGTDVWWKLQSSRKKRCPYSKVQLLELEKEFLYNMYITREQRGEIARKVNLTDRQVKIWFQNRRMKMKRMKQRHEEEA
Bl_Hox13[ACJ74390.1]
  SKNVADGPVVAAADQSVARGGRKKRCPYSKYQLSVLEQEYIQNRYVSRETRLELSQRLNLTDRQVKIWFQNRRMKQKRLEFRSGNQT
Bl_Hox14[ACJ74393.1]
  ATKPAETIAHQNGGGSLTKPVRPKRRPYSKYQLNELENEYVQNQYISRDKRLQLSQKLNLTERQVKIWFQNRRIKQKKLDRRNSEMC
Bl_Hox15[ACJ74394.1]
  VTGGTKGFTTTAGTTTQRPRTRKKRRPYSKPQLALLEDEYASQKFLTKEKRKEISESSSLSERQVMIWFQNRRMKEKKLARRAAAQR
```
