# Supplementary figures and images for: Hox gene cluster of the ascidian, Halocynthia roretzi, reveals multiple ancient steps of cluster disintegration during ascidian evolution
Source: Zoological Lett. 2017 Sep 15;3:17. doi: 10.1186/s40851-017-0078-3 (PMC5602962; doi:10.1186/s40851-017-0078-3)

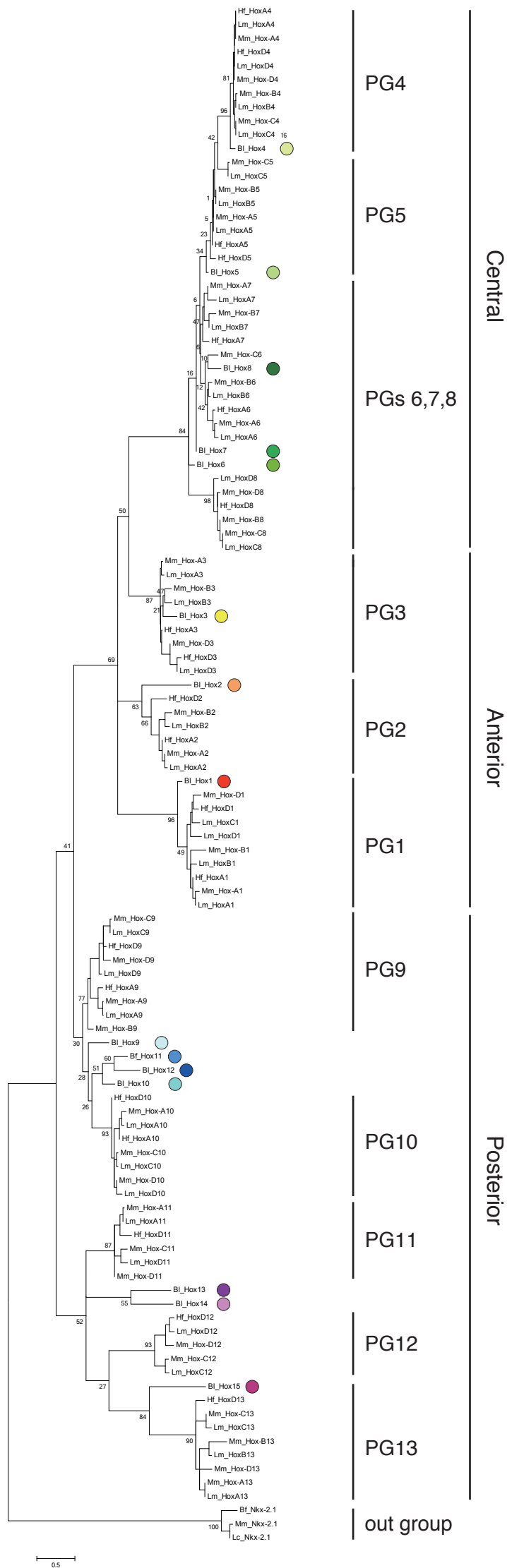

Supplement: Supplementary file 2 — Phylogenetic analysis of amphioxus Hox genes by constructing an ML tree. The ML tree was constructed using homeodomain sequences and the adjacent 20 N-terminal and seven C-terminal amino acids (Additional file 1: Figure S1) and MEGA5 software. The percentage of 1000 replicated trees, in which gene clustering was supported, is indicated at nodes. Within a clade consisting of only vertebrate Hox genes, the percentage was not indicated at the node. Amphioxus Hox genes are marked by colored circles. Color code and taxonomic abbreviations are the same as in Fig. 1, except that the color code for posterior Hox genes is the same as that shown in Fig. 5. Bl and Bf denote Branchiostoma lanceolatum and Branchiostoma floridae, respectively. (PDF 519 kb) [file 40851_2017_78_MOESM2_ESM.pdf]

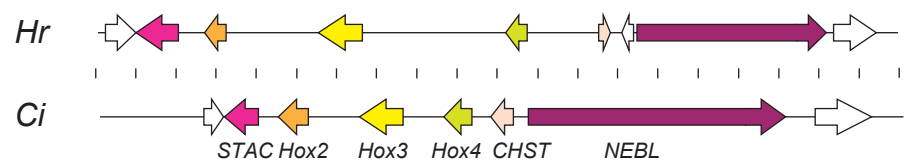

Supplement: Supplementary file 3 — Conservation of gene arrangements surrounding Hox2, 3, and 4 between Hr and Ci. Genomic regions surrounding Hox2, Hox3, and Hox4 are depicted schematically, based on genomic browser information from the ANISEED database (Halocynthia roretzi MTP 2014, Ciona intestinalis type A (KH2012)). Genes are indicated by thick arrows. The color code for Hox genes is the same as in Fig. 4. Pink arrows downstream of Hox2 indicate the gene encoding SH3 and cysteine-rich domain-containing protein (STAC). Pale pink arrows upstream of Hox4 indicate carbohydrate sulfotransferase (CHST1)/chondroitin 6-O-sulfotransferase (C6ST). Dark pink arrows indicate the NEBL gene encoding the Nebullete protein. Blank arrows indicate genes without positional conservation. Grey arrays of short vertical bars indicate 10 kbp. (PDF 246 kb) [file 40851_2017_78_MOESM3_ESM.pdf]
